# ABCB1 haplotypes are associated with P-gp activity and affect a major molecular response in chronic myeloid leukemia patients treated with a standard dose of imatinib

**DOI:** 10.3892/ol.2014.1857

**Published:** 2014-02-07

**Authors:** DOUGLAS VIVONA, LUCIENE TEREZINA LIMA, ALICE CRISTINA RODRIGUES, CAROLINA TOSIN BUENO, GREYCE KELLY STEINHORST ALCANTARA, LUIZA SALDANHA RIBEIRO BARROS, VANIA TIESTSCHE DE MORAES HUNGRIA, CARLOS SÉRGIO CHIATTONE, MARIA DE LOURDES LOPES FERRARI CHAUFFAILLE, ELVIRA MARIA GUERRA-SHINOHARA

**Affiliations:** 1 Department of Clinical and Toxicological Analyses, Faculty of Pharmaceutical Science, University of São Paulo, São Paulo 05508-900, Brazil; 2 Department of Pharmacology, Institute of Biomedical Science, University of São Paulo, São Paulo 05508-900, Brazil; 3 Department of Clinical, Toxicological and Bromatological Analyses, Faculty of Pharmaceutical Science, University of São Paulo, Ribeirão Preto 14040-903, Brazil; 4 Department of Hematology and Hemotherapy, Santa Casa Medical School, São Paulo 01223-001, Brazil; 5 Department of Clinical and Experimental Oncology, Federal University of São Paulo, São Paulo 04023-900, Brazil

**Keywords:** imatinib mesylate, chronic myeloid leukemia, ABCB1

## Abstract

Despite the high efficacy of imatinib mesylate (IM) treatment for chronic myeloid leukemia (CML) patients, some individuals develop resistance due to impaired bioavailability. It has been previously demonstrated that the haplotypes for ATP-binding cassette subfamily B member 1 (*ABCB1*)with c.1236C>T, c.3435C>T and c.2677G>T/A polymorphisms markedly affect the secondary structure of ABCB1 mRNA and its activity. These modifications may affect efflux transporter activity and response to treatment with IM. The aim of the present study was to investigate the influence of ABCB1 haplotypes on P-glycoprotein (P-gp) activity, IM plasma levels and IM response. In total, 28 chronic-phase CML patients treated with a standard dose of IM (400 mg/day) were studied. The patients were selected according to the haplotypes of *ABCB1,* with c.1236C>T, c.3435C>T and c.2677G>T polymorphisms, and were classified into two groups based on the presence of the mutated allele in each genotype for the three *ABCB1* polymorphisms. In addition, expression of P-gp and breakpoint cluster region-abelson 1 (BCR-ABL1), ABCB1 and solute carrier family 22 member 1 (SLC22A1) mRNA were evaluated. The P-gp activity in the wild-type group was found to be higher than that in the mutated group (59.1 vs. 38.3%; P=0.001). Furthermore, the patients who did not achieve major molecular response (MMR) showed a higher rate of efflux mediated by P-gp when compared with individuals who achieved MMR (64.7 vs. 45.7%; P=0.001). All patients without MMR demonstrated effluxes of >60%. In addition, patients without MMR exhibited lower plasma concentrations of IM compared with those with MMR (0.51 vs. 1.42 μg/ml; P=0.001). Higher levels of SLC22A1 mRNA were observed in patients who achieved MMR and complete molecular response (P<0.05). In conclusion, the *ABCB1* 1236CT/3435CT/2677GT and 1236TT/3435TT/2677TT haplotypes are associated with reduced P-gp activity and MMR in chronic-phase CML patients treated with a standard dose of IM.

## Introduction

Imatinib mesylate (IM) is a first-generation tyrosine kinase inhibitor used in the treatment of chronic myeloid leukemia (CML), gastrointestinal stromal tumors and other types of cancer (1). In CML, IM inhibits the oncoprotein breakpoint cluster region-abelson 1 (BCR-ABL1) from phosphorylating subsequent proteins and initiating the signaling cascade necessary for CML development (2). The long-term follow-up results of the International Randomized Study of Interferon and STI-571 trial have confirmed the benefits of IM and the durable response of the drug. Following eight years of IM treatment, no disease progression was identified in patients who achieved complete cytogenetic response (CCyR) and major molecular response (MMR) (3). Therefore, achieving and maintaining such responses are essential for the survival of CML patients treated with IM.

Despite the high efficacy of IM treatment in CML, some patients fail to achieve optimal response. Mutations in the kinase domain of BCR-ABL1 are the main causes of resistance to IM treatment. The mutations interfere with the binding of IM to its target, which subsequently decreases the efficacy of the drug (4). However, accumulating data have indicated a contributing role of pharmacokinetics in IM efficacy, as well as in the initial therapeutic response and time to progression. Variations in drug uptake and efflux transporter activity may affect IM absorption, distribution and excretion, thereby influencing the pharmacokinetics (5).

Several previous studies have demonstrated that IM is a substrate of membrane transporters, such as the ATP-binding cassette subfamily B member 1 [ABCB1; also termed P-glycoprotein(P-gp) and multidrug resistance protein 1 (MDR1)] and solute carrier family 22 member 1 [SLC22A1; also termed human organic cation transporter type 1 (hOCT1)] (6–8). The ABCB1 gene is located in the 7q21.1 chromosome (9) and encodes a glycoprotein of 170 KDa (P-gp and MDR1) (10). The structure of P-gp is comprised of a transmembrane domain with six hydrophobic segments and a cytoplasmic domain with a binding site for ATP, which is responsible for the ATP-dependent movement of a wide variety of xenobiotics (including drugs), lipids and metabolic products across the plasma and intracellular membranes (11). Since P-gp is involved in the efflux of a broad spectrum of drugs, variations in protein expression or activity may affect the pharmacokinetics of IM by reducing or increasing its bioavailability.

The ABCB1 gene is highly polymorphic and >1,200 single nucleotide polymorphisms (SNPs) have been identified within this gene, of which 50 are found within the coding region (12). Three of these SNPs have been particularly investigated: Two silent polymorphisms, c.1236C>T and c.3435C>T; and one polymorphism in the promoter region, c.2677G>T/A, which results in two different amino acid changes (Ala893Ser/Thr). These polymorphisms are in strong linkage disequilibrium and form the three most common haplotypes (1236CC/3435CC/2677GG, 1236CT/3435CT/2677GT and 1236TT/3435TT/2677TT) (13). In the cell lines expressing wild-type and mutant haplotypes with c.1236C>T, c.3435C>T and c.2677G>T/A polymorphisms, no alterations have been previously observed in the mRNA expression of ABCB1 and P-gp protein length. However, it has been demonstrated that haplotypes containing the mutated alleles show major structural modifications that result in changes in the conformation of the binding sites and a subsequent decrease in P-gp activity (12–14).

In our previous study, the *ABCB1* 1236CT/3435CT/2677GT haplotype was found to correlate with MMR in patients responsive to a standard dose of IM (15). However, the haplotype formed by the c.1236C>T, c.3435C>T and c.2677G>T/A polymorphisms in the ABCB1 gene may be associated with the variability of responses to IM due to changes in the functionality of the P-gp efflux pump. Therefore, the aim of the present study was to evaluate the functional activity of P-gp in order to assess the effect of the *ABCB1* 1236CC/3435CC/2677GG, 1236CT/3435CT/2677GT and 1236TT/3435TT/2677TT haplotypes on the efflux capacity mediated by P-gp. In addition, the possible impact on the plasma concentration of IM and molecular responses in patients with CML treated with a standard dose of IM (400 mg/day) were investigated.

## Materials and methods

### Subjects

In total, 28 patients with chronic-phase CML were selected according to the haplotypes of *ABCB1* with c.1236C>T, c.3435C>T and c.2677G>T polymorphisms at the Santa Casa Medical School and Hospital Brigadeiro (both São Paulo, Brazil). A total of 10 patients were identified with the 1236CC/3435CC/2677GG haplotype of *ABCB1* and placed into the wild-type group and 18 patients were identified as carriers of haplotypes with at least one mutated allele in each genotype of the three previously described *ABCB1* polymorphisms (10 patients with the 1236CT/3435CT/2677GT haplotype and eight patients with the 1236TT/3435TT/2677TT haplotype) were placed into the mutated group. The patients were matched for the duration of IM treatment. In addition, all patients were in the chronic phase of CML, were treated with a standard dose of IM (400 mg/day) for an average time period of 60.3±12.6 months and achieved CCyR.

The study protocol was approved by the Investigational Review Board of the University of São Paulo (São Paulo, Brazil), the Hospital Brigadeiro and the Santa Casa Medical School. Written informed consent was obtained from all participants.

### Analysis of ABCB1 polymorphisms

Genomic DNA was isolated from the peripheral blood using a QIAamp DNA blood mini kit (PreAnalytiX GmbH, Feldbachstrasses, Switzerland) according to the manufacturer’s instructions. *ABCB1* genotyping was performed using quantitative polymerase chain reaction (qPCR) followed by restriction fragment length polymorphism analysis, as described previously for the c.3435C>T, c.2677G>T/A (16) and c.1236C>T (17) polymorphisms. The accuracy of the genotyping was evaluated by performing a duplicate analysis of 20% of the randomly selected samples. Furthermore, 10% of the genotypes were confirmed by DNA sequencing.

### Isolation of peripheral blood mononucleated cells (PBMCs)

Isolation of the PBMCs was performed by collecting peripheral blood in Vacutainer^®^ CPT™ tubes (BD Biosciences, Franklin Lakes, NJ, USA). The tubes were inverted five times and centrifuged at 1,720 × g for 20 min at room temperature. Following centrifugation, PBMCs (monocytes and lymphocytes) remain above the gel barrier and, thus, were suspended in the plasma by inverting the tubes. Next, the tubes were centrifuged at 300 × g for 10 min at room temperature, the supernatant was discarded and the pellet was suspended in phosphate-buffered saline (PBS).

### Isolation of mRNA and cDNA synthesis

PBMC mRNA was extracted using a RNeasy mini kit (PreAnalytiX GmbH) according to the manufacturer’s instructions. The concentration and purity of the isolated mRNA was measured using Nanodrop™ ND-1000 (Nanodrop Technologies, Wilmington, ME, USA) and the cDNA was synthesized from 500 ng of RNA using the High-Capacity RNA-to-cDNA™ kit (Invitrogen Life Technologies, Carlsbad, CA, USA).

### BCR-ABL1 mRNA levels

*BCR-ABL1* transcripts were measured by qPCR, as previously described (18). MMR and complete molecular response (CMR) were defined as a reduction of *BCR-ABL1* transcript levels to <0.1 and 0.0032%, respectively, in the peripheral blood samples which were standardized according to the international scale (4).

### ABCB1 and SLC22A1 mRNA expression

The mRNA expression of ABCB1 and SLC22A1 was determined by qPCR using TaqMan™ assays (Hs00184500_m1 and Hs00427552_m1, respectively; Applied Biosystems, Inc. Foster City, CA, USA). A total of five genes (*B2M*, *GAPDH, HMBS*, *HPRT1* and *SDHA*) were analyzed using geNorm™ software (Biogazelle, Zwijnaarde, Belgium)(19) and GAPDH was selected as the reference gene. The relative quantification value of each gene was analyzed using a comparative CT method. The following formula was used to calculate the relative level of transcripts in the sample, normalized to the reference gene, GAPDH: Gene expression = 2^−ΔCT^, where ΔCT = CT of the gene of interest - CT of the reference gene.

All reactions were duplicated and each reaction plate was performed without a sample as a negative control to assess the possible contamination of reagents.

### Flow cytometric assessment of P-gp activity [rhodamine 123 (Rh123) efflux]

P-gp functional activity was assessed by the ability of cells to induce the cellular efflux of Rh123, a P-gp substrate, using flow cytometry (20). The PBMCs (1×10^6^) were incubated with Rh123 (1 μM) in PBS at 37°C for 15 min in the dark. The cells were then incubated in the presence and absence of a P-gp inhibitor, PSC-833 (50 μM), for 1 h at 37°C. At 0 and 60 min of incubation, cells were washed twice with PBS. Next, the cell pellet was suspended in 300 μl PBS and immediately used for the flow cytometric analysis of Rh123 retention. The Rh123 fluorescence was determined by flow cytometry using the FACSCanto II flow cytometer (BD Biosciences) at a wavelength of 525 nm. Lymphocytes were gated by forward and side scatter, excluding cell debris and other blood cells. In total, 10,000 cells were counted in each sample. The P-gp mediated efflux was calculated using the following formulae: Rh123 efflux (%) = ([Rh123] at 0 min - [Rh123] at 60 min)/[Rh123] at 0 min; and P-gp mediated efflux (%) = Rh123 efflux in the absence of PSC-833 (%) - Rh123 efflux in the presence of PSC-833 (%).

### Determination of P-gp expression

The PBMCs of all patients were tested for P-gp expression, which was quantified using mouse anti-human P-gp monoclonal antibody (17F9; Santa Cruz Biotechnology Inc., Santa Cruz, CA, USA). The protocol was adapted from that of Rodrigues *et al* (21). Briefly, the PBMCs (1×10^6^) were washed with PBS and fixed with 1% formaldehyde for 15 min at room temperature. Following one wash with PBS, the cells were incubated with the primary anti-human monoclonal P-gp antibody, 17F9 (1:50 dilution in PBS), overnight at 4°C. Following incubation, the cells were washed with PBS and incubated with the secondary antibody (1:50 dilution in PBS), goat anti-IgG antibody conjugated with fluorescein isothiocyanate (FITC), for 40 min at 4°C. Following incubation, the cells were suspended in 300 μl PBS for flow cytometric analysis using the FACSCanto II flow cytometer (BD Biosciences). Lymphocytes were gated as previously described and a total of 10,000 cells were counted in each sample. As a negative control, PBS was used instead of the primary antibody.

The results were expressed as the ratio between the mean fluorescence intensity (MFI) of cells treated with the 17F9 primary antibody and FITC-labeled secondary antibody, divided by the MFI of cells treated with the secondary antibody only (negative control). Values >1.1 were considered positive for P-gp expression (20).

### Imatinib plasma levels

Blood samples were collected 24 h (±2 h) following the administration of the IM dose, according to the instructions of Larson *et al* (22). Plasma concentrations of IM and its metabolite, CGP74588, were measured using capillary electrophoresis with lidocaine (Sigma-Aldrich, St. Louis, MO, USA) as the internal standard, according to the instructions of Ajimura *et al* (23), with minor modifications. The limit of quantification was 0.125 μg/ml, and the precision and accuracy (coefficient of variation) at concentrations between 0.125 and 5.00 μg/ml were >15%.

### Statistical analysis

The database and statistical analysis were performed using the SPSS software, version 17.0 (SPSS, Inc., Chicago, IL, USA) and graphs were obtained with GraphPad, version 5.04 (GraphPad Software, San Diego, CA, USA). Numerical variables were compared using the Student’s t-test or Mann-Whitney U test. Comparison of categorical variables was performed by the χ^2^ or likelihood ratio tests. The non-parametric Mann-Whitney U test was used to evaluate the differences in the mRNA expression of the ABCB1 and SLC22A1 genes, IM plasma levels and P-gp activity among the groups with different haplotypes. Spearman’s correlations were conducted to assess the associations between the patient variables (P-gp activity, P-gp expression, ABCB1 and SLC22A1 mRNA expression, BCR-ABL1 transcripts and IM plasma levels). Values >0.7 were considered to indicate a strong correlation, 0.3–0.7 a moderate correlation and <0.3 a weak correlation. P<0.05 was considered to indicate a statistically significant difference.

## Results

### Clinical results

The patients included in the present study had similar distributions in age, blood cell counts (erythrocytes, leukocytes and platelets), time of diagnosis, treatment prior to IM and initiation of IM treatment (P>0.05), according to the different haplotype groups. All patients achieved complete hematologic response and CCyR (Table I). In addition, the frequencies of MMR and CMR in the wild-type and mutated groups were comparable (80 vs. 88.9% for MMR; and 20 vs. 17.7% for CMR; P>0.05).

### P-gp activity and IM response

**Figure 1:**
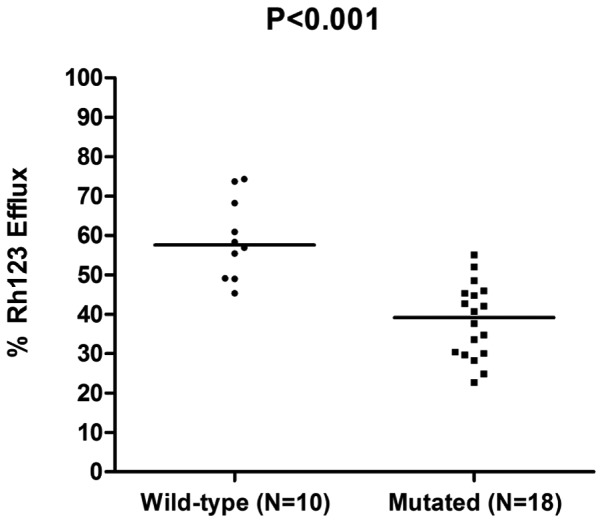
Effect of ATP-binding cassette subfamily B member 1 haplotypes on P-gp activity. P-gp activity was measured as a percentage of the Rh123 efflux and the medians were compared using the Mann-Whitney U test. The haplotype groups were as follows: Wild-type, 1236CC/3435CC/2677GG; and mutated, 1236CT/3435CT/2677GT and 1236TT/3435TT/2677TT. P-gp, P-glycoprotein; Rh123, rhodamine 123.

**Figure 2:**
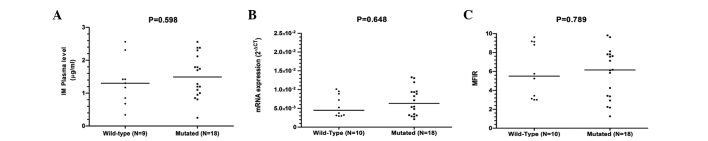
(A) IM plasma levels, (B) ATP-binding cassette subfamily B member 1 mRNA expression and (C) P-glycoprotein expression between the wild-type and mutated groups. The medians were compared using the Mann-Whitney U test. The haplotype groups were as follows: Wild-type, 1236CC/3435CC/2677GG; and mutated, 1236CT/3435CT/2677GT and 1236TT/3435TT/2677TT. IM, imatinib mesylate; MFIR, mean fluorescence intensity ratio.

The median Rh123 efflux in the wild-type and mutated groups was 59.1 (range, 54.8–69.5) and 38.3 (range, 27.4–47.9), respectively. A higher rate of P-gp activity was observed in patients carrying the wild-type haplotype compared with those carrying the mutated allele (P<0.001; Fig. 1). The different haplotypes showed no influence on ABCB1 mRNA expression, P-gp expression and IM plasma levels (Fig. 2).

A strong and direct correlation was identified between ABCB1 mRNA expression and P-gp expression (r=0.747; P=0.001). In addition, P-gp activity was found to positively and moderately correlate with *BCR-ABL1* transcript levels (r=0.570; P=0.001), whereas SLC22A1 mRNA expression was found to negatively and moderately correlate with *BCR-ABL1* transcript levels (r=-0.407; P=0.032).

The patients who did not achieve MMR showed a higher rate of efflux mediated by P-gp compared with those individuals who achieved MMR (64.7 vs. 45.7%; P=0.001). Furthermore, the individuals who achieved MMR and CMR had a higher median rate of SLC22A1 mRNA expression when compared with individuals who did not achieve MMR and CMR (P<0.05). All patients without MMR showed effluxes of >60%. However, no association was found between P-gp activity and CMR (Table II). Additionally, patients without MMR exhibited lower plasma concentrations of IM when compared with those who achieved MMR (0.51 vs. 1.42 μg/ml; P=0.001). However, no association was observed between the plasma concentrations of IM and CMR (Table II).

## Discussion

The *ABCB1* c.1236C>T, c.3435C>T and c.2677G>T/A polymorphisms have been associated with cancer risk, as well as the variability of response to chemotherapy treatments (24,25). It has been previously demonstrated that the three polymorphisms are in strong linkage disequilibrium. Considering the various haplotypes of *ABCB1* with c.1236C>T, c.3435C>T and c.2677G>T/A polymorphisms, it has been reported that ~60% of the population, regardless of ethnicity, carry at least one of the following three haplotypes: 1236CC/3435CC/2677GG, 1236CT/3435CT/2677GT and 1236TT/3435TT/2677TT (26).

Dulucq *et al* (27) previously observed that CML patients treated with a standard dose of IM (400 mg/day) and who were carriers of the 1236C/3435C/2677G haplotype achieved MMR less often than patients carrying other haplotypes (44.6 vs. 70%). In addition, the authors demonstrated that the 1236T/3435T/2677T haplotype was associated with MMR and serum concentrations of IM >1.0 μg/ml.

In the present study, patients who did not achieve MMR were observed to exhibit a higher P-gp retention (measured by Rh123 efflux), which was associated with the wild-type haplotype (1236CC/3435CC/2677GG). Previous studies have also associated P-gp activity with the ABCB1 haplotypes (12,14) and, subsequently, the 1236CC/3435CC/2677GG haplotype has been associated with failure of treatment with antiepileptic drugs (28). An additional study that investigated patients with various types of cancer, including CML, demonstrated that the rate of elimination of the P-gp substrate, sestamibi (99mTc), was higher in individuals who carried the 3435CC/2677GG haplotype, suggesting a higher P-gp activity in such patients (29). Other studies have shown that subjects with the 1236TT/3435TT/2677TT haplotype have a higher plasma concentration of digoxin, simvastatin, atorvastatin and anthracycline compared with subjects carrying the other haplotypes (30–33). Recently, the 1236T/3435T/2677T haplotype has been associated with an improved response to treatment with anthracyclines in patients with breast cancer (33). In a previous study, an increased serum concentration of digoxin was found in 195 patients with congestive heart failure carrying the 1236TT/3435TT/2677TT haplotype (30). Furthermore, a comparable effect was observed in Chinese patients treated with digoxin (32). Keskitalo *et al* (31) also showed that individuals carrying the 1236T/3435T/2677T haplotype had a higher plasma concentration of simvastatin and atorvastatin.

By contrast, Kim *et al* (34) showed that carriers of the 1236CC/3435CC/2677GG haplotype exhibited ~40% higher area under the plasma level-time curve values for fexofenadine compared with individuals carrying the heterozygous and mutated haplotypes. However, other studies have found no correlation between the c.1236C>T, c.3435C>T and c.2677G>T/A polymorphisms and the pharmacokinetics of aliskiren, methadone, nortriptyline, docetaxel and paclitaxel (35–37).

These contrasting results suggest that the influence of P-gp transport may be specific for each substrate. To date, the mechanisms involving the drug structure and the interaction with P-gp have not yet been elucidated. Furthermore, the majority of studies have evaluated their results according to the associations observed between the phenotype and genotypes of the c.1236C>T, c. 3435C>T and c.2677G>T/A polymorphisms. However, these studies have not performed P-gp activity tests to confirm whether these genotypes are associated with changes in the rate of the P-gp efflux pump or whether the transporter alone is involved in the response or change in drug concentration.

The results of the current study are consistent with results previously presented by Dulucq *et al* (27), and Vivona *et al* (15) revealed an association between the presence of at least one mutant allele in the three previously described polymorphisms and MMR. The lower efflux mediated by the ABCB1 haplotypes suggests that reduced P-gp activity is associated with higher IM intracellular concentrations, which is likely to result in MMR.

IM plasma levels may affect the response to this drug and, subsequently, it has been suggested that the IM concentration of 1.0 μg/ml is optimal for achieving MMR (38–40). Notably, in the present study, patients who achieved MMR had higher IM plasma concentrations compared with those without MMR (1.42 vs. 0.51 μg/ml); however, no association was observed between the IM plasma levels and CMR. In addition, all patients included in the present study achieved CCyR, even with IM concentrations <1.0 μg/ml. These results are similar to those of two previous studies in which no correlation was identified between the IM plasma concentration and CCyR (39,40).

Conflicting results regarding the association of the c.1236C>T, c.3435C>T and c.2677G>T/A polymorphisms with the elimination of IM, as measured by renal clearance, have been observed in certain previous studies (40–42). A study conducted on 34 CML patients that evaluated the effect of the *ABCB1* c.3435C>T polymorphism on IM pharmacokinetics showed that individuals carrying the 3435CC genotype had a higher renal clearance of IM than individuals carrying the 3435CT and 3435TT genotypes (41). By contrast, an additional study on 22 CML patients showed that carriers of the 1236TT/3435TT/2677TT haplotype had a higher renal clearance of IM compared with patients carrying the other haplotypes (42). However, in this study, only four patients were carriers of the 1236TT/3435TT/2677TT haplotype. In the present study, no correlation was found between the 1236CC/3435CC/2677GG, 1236CT/3435CT/2677GT and 1236TT/3435TT/2677TT haplotypes and IM plasma concentration. These results are consistent with an additional study that found no correlation between the c.1236C>T, c.3435C>T and c.2677G>T/A polymorphisms and IM plasma levels (40). These results suggest the requirement for further studies with a larger number of individuals to characterize the correlations between c.1236C>T, c.3435C>T and c.2677G>T/A polymorphisms and the pharmacokinetics of IM.

Previous studies analyzing the manner in which the c.1236C>T, c.3435C>T and c.2677G>T/A polymorphisms alter the interaction between P-gp and its substrates are limited. Several studies have shown that each substrate can interact with different binding sites of P-gp (43,44); however, it is difficult to determine in advance which binding sites are important for each drug class. Fung and Gottesman (12) used the UIC2 monoclonal antibody directed against the extracellular epitope of P-gp *in vitro* and demonstrated that the conformation of the wild-type haplotype for the c.1236C>T, c.3435C>T and c.2677G>T/A polymorphisms is different from that of the mutated haplotypes. This change was demonstrated to be dependent on the presence of the c.3435C>T polymorphism in the haplotype. This study suggested that synonymous mutations at the ABCB1 haplotype produce a subtle change in the substrate binding site.

The changes in protein function caused by synonymous *ABCB1* polymorphisms may not be explained by the amino acid sequence of the protein. However, the *in vitro* and *in vivo* observations from previous studies suggest that the c.3435C>T polymorphism affects protein folding and function (13,45) when it appears in a haplotype with the c.1236C>T and c.2677G>T/A polymorphisms. Tsai *et al* (46) suggested that the synonymous c.3435C>T polymorphism causes the ribosome to pause in the reading of codons, which subsequently affects protein translation.

In the present study, the reduced P-gp activity in patients carrying the heterozygous and mutated haplotypes compared with those carrying the wild-type haplotype may be explained by a possible conformational change in the carrier, but not by changes in the expression of the ABCB1 mRNA or P-gp expression. This modification leads to a decreased efflux of IM, which changes the intracellular concentration of IM. The results of the present study suggest that changes in the intracellular concentration may be crucial for MMR, although all patients included achieved complete hematologic response and CCyR. The achievement of molecular responses appears to be a complex process, which is dependent on higher intracellular concentrations of IM and altered pharmacokinetics associated with low SLC22A1 mRNA expression and hOCT1 activity. Additionally, increased efflux associated with the presence of the *ABCB1*1236CC/3435CC/2677GG haplotype may substantially impact the molecular response to IM treatment.

The aim of the present study was to assess the expression of SLC22A1 mRNA in order to evaluate the possible combined action of SLC22A1 with P-gp activity. A higher expression of SLC22A1 mRNA was observed in patients with MMR and CMR compared with those without molecular response. The results are consistent with the results of a previous study that found higher levels of *SLC22A1* transcripts in patients with MMR and CMR following six years of IM treatment (39).

In conclusion, the present study demonstrated that the *ABCB1* 1236CT/3435CT/2677GT and 1236TT/3435TT/2677TT haplotypes are associated with lower P-gp activity and MMR in chronic-phase CML patients treated with a standard dose of IM (400 mg/day). However, additional factors, such as other pharmacokinetic and genetic polymorphisms in the ABCB1 and SLC22A1 genes, and the expression of metabolizing enzymes (such as CYP3A4 and CYP3A5) and other carriers (for example SLCO1A2 and ABCA3), may interact and explain the variability of the IM response. Prospective studies involving larger numbers of patients and numerous pharmacogenetic markers are required to definitively elucidate the influence of these factors on the pharmacokinetics of IM.

## Figures and Tables

**Table I:** Distribution of a number of variables between the CML patients.

	Haplotype		
		
Variables	Wild-type (n=10)	Mutated (n=18)	P-value
Age^a^	51.2 (30.2–57.0)	53.7 (33.8–68.0)	0.627
Erythrocytes (x10^6^/mm^3^)^a^	4.59 (4.21–5.12)	4.3 (3.98–5.10)	0.885
Leukocytes (x10^3^/mm^3^)^a^	5.10 (4.30–5.80)	4.22 (3.92–6.57)	0.797
Platelets (x10^3^/mm^3^)^a^	200 (180–237)	193 (178–248)	0.795
Time of diagnoses (months)^a^	62.5 (59.7–100)	65.5 (59.0–109.2)	0.832
Preview treatment to IM^b^			
Interferon-α	4 (40.0)	9 (50.0)	0.879
None	6 (60.0)	9 (50.0)	
Time period of IM use (months)^a^	59.5 (53.7–72.0)	60.5 (52.1–75.3)	0.901

Data are presented as the ^a^mean (95% confidence interval) and ^b^absolute frequency (relative frequency). Student’s t-test was used for comparing the means of the wild-type and mutated groups. Wild-type, 1236CC/3435CC/2677GG; mutated, 1236CT/3435CT/2677GT and 1236TT/3435TT/2677TT; CML, chronic myeloid leukemia; IM, imatinib mesylate.

**Table II:** Correlations between P-gp activity, ABCB1 and SLC22A1 mRNA expression, P-gp expression and IM plasma levels in individuals with or without MMR and CMR.

	MMR			CMR		
				
	Yes (n=24)	No (n=4)	P-value^b^	Yes (n=5)	No (n=23)	P-value^b^
P-gp activity	45.7 (32.7–57.2)	64.7 (47.5–73.2)	0.001	45.1 (29.1–59.3)	46.0 (31.3–62.8)	0.890
ABCB1 mRNA	5.31 (3.31–9.47)	8.41 (4.41–9.27)	0.205	8.33 (3.20–10.10)	6.41 (2.11–9.12)	0.447
P-gp expression^a^	6.0 (3.0–7.6)	6.4 (2.3–8.1)	0.989	6.6 (3.0–8.6)	5.7 (2.3–9.0)	0.713
SLC22A1 mRNA	0.95 (0.68–1.59)	0.54 (0.43–0.82)	0.042	1.80 (1.40–2.90)	0.81 (0.40–1.10)	0.001
IM plasma levels, μg/ml	1.42 (1.11–2.12)	0.51 (0.27–1.01)	0.001	1.56 (1.25–1.73)	1.28 (0.85–2.30)	0.753

a Mean fluorescence intensity ratio and

b Mann-Whitney U test.

Data are presented as the median (interquartile range, 25–75th percentile). MMR is defined as *BCR-ABL1* transcript levels <0.1; and CMR is defined as *BCR-ABL1* transcript levels <0.0032. For an improved representation of the mRNA expression, the values were multiplied by 1,000. P-gp, P-glycoprotein; ABCB1, ATP-binding cassette subfamily B member 1; SLC22A1, solute carrier family 22 member 1; IM, imatinib mesylate; MMR, major molecular response; CMR, complete molecular response.
